# Can Telemedicine Optimize the HCV Care Cascade in People Who Use Drugs? Features of an Innovative Decentralization Model and Comparison with Other Micro-Elimination Strategies

**DOI:** 10.3390/biology11060805

**Published:** 2022-05-24

**Authors:** Riccardo Nevola, Valerio Rosato, Vincenza Conturso, Pasquale Perillo, Teresa Le Pera, Ferdinando Del Vecchio, Davide Mastrocinque, Annalisa Pappalardo, Simona Imbriani, Augusto Delle Femine, Alessia Piacevole, Ernesto Claar

**Affiliations:** 1 Hepatology Unit, Ospedale Evangelico Betania, 80147 Naples, Italy; valeriorosato@gmail.com (V.R.); pasqualeperillo@hotmail.it (P.P.); davidemastrocinque4@gmail.com (D.M.); annalisa.pappalardo88@gmail.com (A.P.); ernestoclaar@gmail.com (E.C.); 2 Internal Medicine Unit, Department of Advanced Medical and Surgical Sciences, University of Campania Luigi Vanvitelli, 80138 Naples, Italy; simo.imbriani@gmail.com (S.I.); gudf96@gmail.com (A.D.F.); alessia0694@gmail.com (A.P.); 3 Service for Addiction, DS32, ASL Napoli 1, 80147 Naples, Italy; enzaconturso@gmail.com (V.C.); teresa.lepera@fastwebnet.it (T.L.P.); ferdelv@libero.it (F.D.V.)

**Keywords:** people who use drugs, PWUD, HCV, telemedicine

## Abstract

**Simple Summary:**

The global fight against the hepatitis C virus (HCV) involves the processes of micro-elimination of populations at risk. People who use drugs (PWUDs) represent a viral reservoir, due to the historical challenge in treating this population. In particular, the difficulties in the linkage to care of these patients, as well as low adherence to therapies and follow-up and the risk of re-infection make PWUDs a “difficult-to-treat” population. In view of this, the testing of effective management and treatment models for chronic HCV infection in PWUDs is crucial for promoting its elimination. Telemedicine could be a successful solution in the integration and decentralization of care services.

**Abstract:**

People who use drugs (PWUDs) are a crucial population in the global fight against viral hepatitis. The difficulties in linkage to care, the low adherence to therapy, the frequent loss to follow-up and the high risk of re-infection make the eradication process of the hepatitis C virus (HCV) really hard in this viral reservoir. Several management and treatment models have been tested with the aim of optimizing the HCV care cascade in PWUDs. Models of decentralization of the care process and integration of services seem to provide the highest success rates. Giving this, telemedicine could favor the decentralization of diagnostic-therapeutic management, key for the implementation of linkage to care, reduction of waiting times, optimization of adherence and results and reduction of the costs. The purpose of this literature review is to examine the role and possible impact of telemedicine in optimizing the HCV care cascade, comparing the different care models that have shown to improve the linkage to care and therapeutic adherence in this special population.

## 1. Introduction

Despite the availability of highly effective and tolerable antiviral therapies, chronic hepatitis C virus (HCV) infection still represents a significant global health problem, accounting for 56.8 million people with active infection and over 250,000 estimated deaths per year [1]. The challenge set by the World Health Organization (WHO) of eliminating HCV by 2030 appears to be a difficult goal to achieve [2]. While only 11 countries appeared in line with this goal by 2019 [3], the depletion of the pool of patients with known HCV infection first and then the advent of the SARS-CoV-2 pandemic resulted in a significant slowdown in virus elimination programs [4]. To date, the identification of HCV cases seems to be the limiting factor towards the objectives suggested by the WHO. For this reason, screening of population groups at high risk of HCV infection is essential [5]. On this matter, people who use drugs (PWUDs) represent a priority population due to the high prevalence of HCV infection, high transmissibility, and treatment challenges. The prevalence of HCV-positivity among PWUDs is estimated to be about 52%, representing a significant reservoir for the virus [6]. In addition to the favorable impact on the health of the individual patient, the treatment of HCV infection in PWUDs has been shown to determine a significant benefit in the spread of the infection in the general population [7]. Therefore, diagnosis and treatment of HCV cases in PWUDs are key steps in the global fight against viral hepatitis.

The prevalence of viral hepatitis is particularly high in this population. In addition, PWUDs are characterized by considerable difficulties in linkage to care, low therapeutic adherence, frequent loss to follow-up, and high risk of re-infection, making the viral eradication process challenging. In order to optimize the HCV care cascade in this population, several management and treatment models have been tested. Recently, Electronic Health (eHealth: remote monitoring, teleconsultation, mobile device–supported care, informative programs), including telemedicine, has been shown to positively impact HCV treatment processes among PWUDs, maximizing results, and could represent a successful weapon in the fight against viral hepatitis [8,9]. In particular, telemedicine helps to manage the diagnosis and antiviral treatment in a decentralized way, improving linkage to care and adherence to therapy, in addition to significantly reducing costs.

The purpose of this narrative review is to define the characteristics and difficulties of treating a special population such as that of PWUDs and to evaluate the role and possible impact of telemedicine in implementing the HCV care cascade, comparing the different models of assistance that allow to optimize linkage to care and therapeutic adherence in this population.

## 2. PWUDs: Characteristics of a Special Population

In the context of HCV infection, PWUDs historically represent a challenging population, due to the difficulties in linkage to care, poor compliance to treatments, loss to follow-up and risk of re-infection. However, due to the high prevalence of chronic viral infections, the eradication of HCV in this population is crucial. It is estimated that there are over 15 million PWUDs globally and more than two thirds are male [6]. In this population, the estimated seropositivity rate for HCV Antibodies (HCV-Ab) is 52.3% (42.4–62.1%), accounting for more than 8 million cases globally [6]. It has been demonstrated that 23% of new diagnoses of HCV infection and 33% of annual mortality rates for HCV are related to PWUDs [10].

The spread of blood-borne viruses among PWUDs occurs via contaminated injection paraphernalia [11]. In particular, PWUDs with a longer history of injection drug use (IDU) in public or outdoor spaces, those with previous arrests, and those who share syringes and paraphernalia are more exposed to the risk of contracting HCV [12]. Prostitution, incarceration, and homelessness status can further increase the exposure of PWUDs to HCV [6,13,14].

Overall, the most frequently HCV genotypes among PWUDs are genotype 1a and genotype 3 [15]. In any case, the distribution of viral genotypes among PWUDs is affected by regional modalities of virus spreading, accounting for the prevalence of genotypes 2 and 6 in Asia and of genotypes 1a and 4 in Africa [15]. Genotype 3, the most frequent in the PWUDs population to date, shows suboptimal response rates to new direct antiviral agents (DAAs). Although the rates are elevated when compared to Interferon (IFN)-based regimens, they are lower when compared to the response of other genotypes, particularly when liver cirrhosis coexists or therapeutic failure has already been experienced [16]. In such cases, a longer duration of antiviral treatment or the co-administration with ribavirin may be needed [17]. Regardless of treatment compliance, the high frequency of genotype 3 in the PWUDs population could potentially be associated with suboptimal sustained virological response (SVR) rates.

The exposure to blood-borne viral infections of PWUDs accounts for high rates of coinfection with the hepatitis B virus (HBV) and human immunodeficiency virus (HIV). It is estimated that the prevalence rates of HCV/HBV and HCV/HIV coinfections in PWUDs are 3% and 13%, respectively, and that 2% of PWUDs are affected by a triple infection (HBV/HCV/HIV) [10]. Co-infection with HBV and/or HIV leads to an increased risk of hepatocellular carcinoma (HCC) and all-cause mortality rates [18,19]. Interactions with other antiviral (HBV and HIV) drugs and the risk of HBV infection to be reactivated makes the treatment even more complex and determines the need for close surveillance and careful follow-up of these patients, who are characteristically poorly compliant [20,21].

The risk of re-infection in this population is due to the frequent persistence of active drug addiction. Data from a recent meta-analysis quantifies this risk in 1.94 per 100 person years (95% CI, 0.87–4.32) for recent PWUDs and in 0.55 per 100 person years (95% CI, 0.17–1.76) for PWUDs in opioid substitution therapy (OST) [22]. In any case, the possibility of re-infection should not discourage the initiation of treatment for HCV infection, since it allows to stop viral transmission in this reservoir.

## 3. HCV Care Cascade in PWUDs

**Figure 1:**
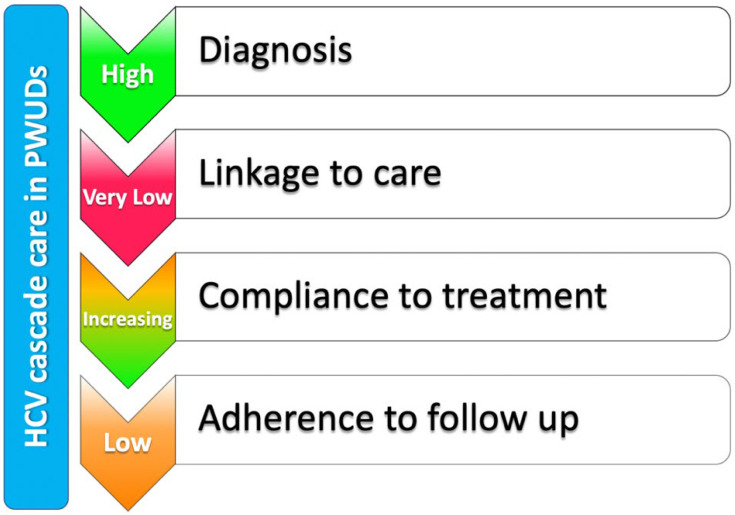
HCV care cascade in the PWUDs population: lights and shadows.

The care cascade of the patient with HCV infection refers to the diagnostic-therapeutic path that starts with diagnosis and goes from linkage to care to treatment and subsequent follow-up. Despite the high rate of diagnosis of HCV infection, in the PWUDs population it is historically challenging to achieve an adequate treatment (linkage to care) and adherence to therapy (compliance) (Figure 1) [23].

In the era of IFN-based therapies, the treatment of chronic HCV infection was not very effective and burdened with numerous side effects, and for these reasons was even more hard in a PWUD. Moreover, the presence of psychiatric adverse events (depression, anxiety, suicide attempts) attributable to the IFN often made HCV-positive PWUDs patients (with frequent psychiatric comorbidities, such as anxiety, depression, tendency to self-harm, and suicidal ideas) not eligible for antiviral treatment. This resulted in an extremely low rate of PWUDs undergoing antiviral treatment (<20%) and in persistence of a rich viral reservoir in this subgroup of the population until today [24,25]. The introduction of therapies (DAAs) that are as effective as they are free from side effects, are exclusively for oral use, and are of short duration has led to an increase in the number of patients eligible for treatment.

Where there is adequate compliance, the effectiveness of antiviral treatment with DAAs is extraordinarily high [24,26], approximating the SVR rates obtained in HCV-positive patients who do not use intravenous drugs. The concomitant use of OST is safe and does not reduce virological response rates [22,27]. Therefore, antiviral treatment is currently indicated in all PWUDs with HCV infection, regardless of the status of active drug addiction, the use of OST, and the risk of re-infection, in order to reduce viral transmission [17,28]. In addition, the manageability of the new antiviral treatment schemes has allowed to significantly improve adherence to therapy [26]. At present, therefore, linkage to care seems to be the weakest step in the care cascade for PWUDs patients with HCV infection (Figure 1). In fact, frequently, the diagnosis of infection is not followed by appropriate treatment or it is started with considerable delay [29]. Among PWUDs, women, young adults and homeless people are in particular the categories of patients with a lower rate of antiviral treatment [30]. Among the obstacles to the treatment of HCV in PWUDs in Italy, the impossibility to prescribe antiviral drugs at public services for addiction (called SerD in Italy), the lack of an effective link between peripheral structures and prescribing centers and fragmentation of care services (diagnostic and therapeutic) represent the most significant barriers to the elimination of the virus in this population.

## 4. Treatment Models

Several models aimed to enhance the efficiency of the HCV care cascade among PWUDs have been evaluated. Overall, integrated assistance and simplified access to services have been shown to implement the number of patients referred for treatment and to improve compliance [23]. In this regard, Messina et al. [31] have demonstrated that creating a direct link between prescribing centers and public services for addiction that are adequately prepared and able to provide all diagnostic tools locally significantly increases the number of diagnoses of active infection and HCV-positive PWUDs initiated for antiviral treatment. Telemedicine can provide an additional opportunity for virtual integration of assistance, optimizing the effectiveness of the care cascade for patients with HCV infection [32]. In fact, telemedicine, through remote patient monitoring, is able to favor the decentralization of diagnostic-therapeutic management, which is the key for the implementation of linkage to care, the optimization of adherence and results, and reduction of the costs [33].

### 4.1. Our Telemedicine-Based Model

**Figure 2:**
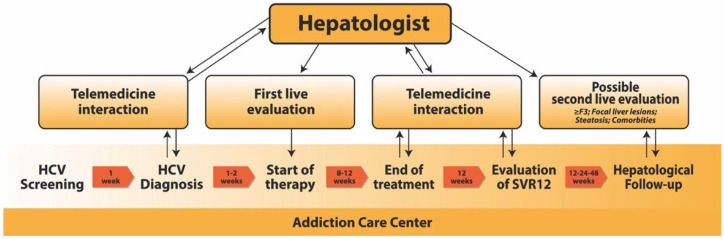
Graphic description of our “patient–centered approach” to HCV treatment in PWUDs.

Our group has recently evaluated a decentralized assistance model, based on the use of telemedicine, with the aim of favoring linkage to care, reducing waiting times to start antiviral treatment, and optimizing the HCV treatment cascade in PWUDs patients (Figure 2) [8,9].

This “patient-centered” model, after the initial screening, provided a complete hepatological evaluation performed directly at the public services for addiction (including medical history, physical examination, blood chemistry, and virological tests) for HCV-Ab positive patients. After the diagnosis performed by SerD physicians and the assessment of comorbidities, drug interactions, and any individual needs by hepatologists in telemedicine, patients were evaluated by a specialist (using abdominal ultrasound and transient elastography) within 3 weeks and started the antiviral treatment immediately after the in-person visit. DAA treatment was tailored to clinical or social features of the patients under the supervision of SerD physicians, also performing a daily administration during outpatient visits in order to maximize adherence. After initiation of treatment, patients were followed by a hepatologist exclusively through telemedicine interaction (telephone or video call), with the possibility to remotely monitor the treatment and to provide necessary support and identify cases that required a specialist visit. The virological response at the end of the treatment and the following 12 weeks was assessed by a HCV-RNA assay performed directly at the SerD and remotely verified by the hepatologist. The subsequent follow-up was personalized in relation to the state of the disease and comorbidities. Generally, specialist follow-up was reserved for patients with advanced liver fibrosis, focal liver lesions, and/or cofactors of liver injury.

Of 690 patients managed by the SerD, 135 of them (19.6%) had active HCV infection. All viraemic patients underwent antiviral treatment (135/135 patients, 100%). The median time from the diagnosis of HCV infection to the start of treatment was 3 weeks. There were 6 cases of drop-out (4.4%) and adherence to therapy was <90% (assessed on the accomplishment of therapy or on the percentage of visits attended) in 9 patients (6.6%). Overall, SVR at 12 weeks post-treatment was achieved in 133 patients (98.5%). Differently from what was expected, 5 out of 6 patients (83.3%) who stopped treatment early still achieved SVR. Further therapeutic failure was recorded among patients who showed poor adherence to treatment. During the subsequent follow-up, only one case of reinfection was reported.

This innovative decentralized management model based on telemedicine has therefore allowed to optimize the linkage to care of viraemic patients leading to maximal adherence to therapy and highest virological response rate. Moreover, the possibility to perform diagnostic tests (blood chemistry, virological, and instrumental) in loco allowed to achieve a “territorial” management of the antiviral treatment, maximizing compliance and follow-up.

### 4.2. Other Telemedicine-Based Models

To our knowledge, only three other studies have evaluated the effectiveness of telemedicine in the setting of the HCV care cascade in the PWUDs population (Table 1) [34,35,36].

Talal et al. [34] demonstrated, in this regard, how the management through biweekly telemedicine sessions of opioid use disorder (OUD) patients with HCV infection is feasible with high rates of virological response. In this study, however, the rate of patients diagnosed with active infection who started antiviral treatment was significantly lower (73.8%) than in our model. This management method seems to be favorably accepted by patients, owing to the convenience, privacy and confidentiality of the meetings [45].

A model of assistance similar to ours (decentralization with the support of telemedicine) was tested by Dhiman et al. (Punjab Model) [35]. The Punjab Model is an interactive model of decentralized services that provides for the management of antiviral treatment by a non-specialist personnel belonging to 25 spoke centers. The personnel are trained in the prescription and management of treatment with DAAs and PWUDs without cirrhosis or with compensated cirrhosis and supervised by telemedicine fortnightly. Despite a high SVR rate (91.1%), this model nevertheless resulted in a high drop-out rate (approximately 20% of patients referred for treatment) and in low compliance at follow-up (available for 86% of eligible patients). The Punjab model and our management model are similar but, differently from ours, the patient management was performed in primary care centers, outside public services for addiction. Although management remains local, we hypothesize that the choice of the location for diagnosis and treatment can influence adherence. In fact, PWUDs patients generally show a particularly close connection with their public service for addiction, which depends on the personal relationship with the physicians of the Center and the need for the prescription and withdrawal of OST. Moreover, these services for addiction are easily accessible to PWUDs as they are often located near to disadvantaged socio-economic areas and do not require any appointment. In this regard, Rinaldi et al. [42] demonstrated how the management of antiviral treatment in services for addiction is related to a lower drop-out rate compared to external management (0.6% vs. 2.8%, respectively-*p* < 0.001) and to a higher SVR rate (96.2% vs. 91.6%, respectively-*p* < 0.001). In fact, management at a service for addiction represents an independent predictor of virological response (OR 2.356-*p* = 0.002). Previously, Radley et al. [46] had also shown how care pathways carried out entirely at the local community pharmacy (where PWUDs receive substitution therapy) determined the optimization of the HCV care cascade through a more effective linkage to care and better adherence to therapeutic and management protocols than conventional care. Therefore, the choice of the different patient management place could explain the significant discrepancy in treatment adherence and follow-up compliance found between our model and the Punjab Model [8,35].

During the COVID-19 pandemic, Sivakumar et al. [36] evaluated a model of care of HCV-positive PWUDs patients based on telemedicine. This model included in-person visits only at time zero and twelve weeks after the end of the treatment in order to verify the virological response. The other needs were managed through tele-health communication between patients, outreach workers, and clinicians. Of the 35 HCV-positive PWUDs evaluated, 31 of them underwent antiviral treatment within 10 days, achieving SVR in 93.5% of cases (29/31). This model has therefore proved to be a valid response to the pandemic, minimizing outpatient visits, but the small sample size does not allow the results to be generalized.

Telemedicine-based models have been used more frequently in other settings to optimize the care cascade for HCV-infected patients [47,48,49,50,51,52,53,54,55,56,57,58,59,60,61]. Recently, independent studies have shown how the support of telemedicine improve the access and management of antiviral treatment in the Department of Corrections, optimizing therapy effectiveness [47,48,49,50,51,52,53]. In this context, in fact, remote assistance is able to remove the intrinsic barriers to prison management, avoiding delays for specialist consultations and logistical problems related to safety (e.g., transfers to hospitals), which would otherwise limit or slow down access to antiviral treatments for prisoners. Furthermore, in this setting, telemedicine has been shown to significantly reduce management and treatment costs compared to usual clinical practice (−30.6%) [48].

Similarly, telemedicine has also been successfully used to implement the HCV care cascade in remote areas, where the usual management and treatment models would have meant significant logistical difficulties, long waiting times, and high costs [54,55,56,57,58].

Furthermore, the support of telemedicine has been shown to be effective in monitoring antiviral treatment in the lockdown phases following the COVID-19 pandemic [59].

Overall, as suggested by a recent systematic review of the literature [62], telemedicine is therefore effective in optimizing the viral hepatitis care cascade and managing its treatment, with virological response rates comparable to or higher than the standard of care (face-to-face) and significantly lower costs [60,62]. It represents a useful support for decentralization, favoring access to care especially when the availability of specialist visits appears limited for managerial reasons (e.g., PWUD, prisoners) or structural ones (e.g., remote areas). However, due to the limited availability of studies aimed at evaluating the impact of telemedicine on the HCV care cascade, the data need to be confirmed in this setting.

### 4.3. Other Treatment Models

#### 4.3.1. Decentralization Models

Recently, a large meta-analysis aimed at evaluating the management and treatment strategies (with IFN-based and IFN-free regimens) of chronic HCV infection among PWUDs has shown how, with equal efficacy, a complete decentralization (testing and treatment at the same site) significantly increases the linkage to care rate (72%) compared to partial decentralization (testing at decentralized site and referral elsewhere for treatment, 53%) or none (47%) [33]. These models, favoring integrated care, allow PWUDs to overcome the difficulties in accessing health systems [63]. Furthermore, task-shifting from a tertiary care center to non-specialist physicians does not seem to be associated with a reduction in viral eradication rates in IFN-free regimens [33]. In decentralization models (complete or partial), telemedicine can be used as a link between the territory and specialized centers.

Although decentralized management seems to increase linkage to care and allow adequate viral eradication rates, these management strategies alone may not ensure adequate adherence to treatment and subsequent follow-up. As mentioned above, Dhiman et al. [35] in fact, despite demonstrating satisfactory SVR rates in PWUDs adhering to treatment and subsequent monitoring, show how a large proportion of patients managed in a decentralized manner (with the support of telemedicine) in primary care centers prematurely interrupt treatment (approximately 20%) or are lost to follow-up (Table 1). Similarly, Wade et al. [41] demonstrated how, although shifting the antiviral treatment of PWUDs with DAAs to primary care increases the rate of patients initiated for treatment (75% vs. 34%, *p* < 0.01) compared with hospital-based specialist care, SVR rates appear significantly lower than expected. From this perspective, management at services for addiction (rather than primary care centers) correlates instead with lower drop-out rates and higher SVR rates [42].

In a view of a complete decentralization, Radley et al. [46] experimented, through a cluster-randomized trial, a model based on the management of treatments by community pharmacies. In this model, the management of the HCV care cascade (from diagnosis to treatment and related follow-up) was completely entrusted to the community pharmacies where the patient was already assisted for OST (similar to the model we tested). Compared to conventional pathways, this management strategy has been able to produce a greater number of diagnoses as well as simplify the initiation and completion of numerous antiviral treatments. A subsequent analysis also demonstrated how SVR rates are comparable to those obtained through other HCV care pathways (conventional hospital care, drug treatment centers, needle exchanges, nurse-led outreach clinics, and prisons) [44].

In the wake of care models characterized by partial decentralization, however, Messina et al. [31] prospectively conducted a study aimed at evaluating the impact of a close collaboration between services for addiction and tertiary centers for the treatment of HCV. In particular, the model included a training phase for personnel operating in services for addiction, in order to improve their knowledge about the management of HCV infection and related treatment, and a fast “territorial” management protocol for the patient, which could minimize specialist visits outside the service for addiction and treatment times. Compared to the previous standard of care, this model resulted in a significant increase in the number of diagnostic tests performed, in the linkage to care, and in the number of antiviral treatments initiated in the PWUDs population. A similar model was also tested by Mangia et al. [43] in another area of Southern Italy. Similarly, the training of public services for addiction staff, the implementation of screening tests and a fast lane for the treatment of HCV-infected PWUDs (including dedicated transport) in the context of partial decentralized management resulted in an extraordinarily high rate of antiviral treatments started (97.8%) and completed (97.3%), as well as SVR rates comparable to non-PWUD HCV-positive patients (98.6%). In this model, the use of the rapid oral HCV-Ab test may have reduced the time needed to start the antiviral treatment.

#### 4.3.2. Integrated Care

Regardless of the location (central or territorial), the integration of the care system significantly simplifies the management of the HCV-positive PWUD patient, reduces treatment times, and increases adherence. The presence of integrated care facilities where the patient is able to complete the diagnostic tests and the subsequent antiviral treatment also reduces the possibility of drop-out and improves the continuum of care [23]. In loco testing, facilitated referral for HCV assessment and addiction, and a multidisciplinary team providing psychiatric services could further increase the proportion of HCV-positive PWUDs who receive antiviral treatment and achieve an SVR, maximizing the outcomes of the HCV care cascade [63,64,65,66]. In the setting of the PWUDs population, the integration of care services provides the best results when decentralized [33], particularly when it get to the level of services for addiction [42]. In fact, since the PWUD population routinely needs different services (e.g., needle and syringe programs, OST), the inclusion of health services that allow a “one-stop shop” model of care can be a key factor for the removal of existing barriers and the success of these strategies [23]. The French approach for the prevention and treatment of chronic HCV infection in PWUDs is based exactly on these principles [67].

#### 4.3.3. Directly Observed Therapy

In order to increase therapeutic adherence, models of direct administration of the treatment were evaluated. In particular, directly observed therapy (DOT) could lead to an increase in therapeutic adherence compared to self-administered treatment [39,40], with similar SVR rates of the standard of care in non-PWUDs patients. However, the available data seem to be conflicting [68]. A meta-analysis of the (limited) data available in literature seems to indicate that DOT among PWUDs demonstrated significantly higher odds of SVR attainment (OR 2.01) compared to standard of care [69].

#### 4.3.4. Peer Support

Peer support is widely recognized in the fight against drug addiction. Its feasibility in the HCV-positive PWUD patient care cascade has been evaluated in some clinical studies. In particular, these methods of support lead to an increase in screening tests and provide a high rate of referral to secondary care (>89%) among marginalized groups [38,70]. If qualified, peer mentors can play a relevant care role, guiding the patient from diagnostic tests to completion of treatment. Overall, peer support interventions seem to have a positive but non-significant impact in optimizing linkage to care and therapeutic adherence [23]. They seem to be useful in terms of customizing the management of the HCV care cascade.

#### 4.3.5. Economic Incentives

In a small pilot study, Norton et al. [37] tested the impact of economic incentives for HCV-positive PWUDs who underwent antiviral treatment, demonstrating a positive impact in linkage to care compared to the standard of care. However, a randomized clinical trial [38] was unable to demonstrate a significant superiority of economic incentives or peer mentor support in the linkage to care of HCV/HIV co-infected patients. Therefore, economic incentives could favor linkage to care, but do not guarantee therapeutic continuity and should be considered exclusively in addition to other management models.

## 5. Conclusions

In the cascade of care of PWUDs patients with HCV, linkage to care and adherence to treatment need to be implemented. The integrated assistance and the cooperation among the assistance services allow to optimize HCV treatment pathways for PWUDs. Successful models share the integration of services and decentralization. In particular, it would be desirable for the diagnosis and treatment processes to be carried out in the context of addiction services already attended by this population. In this view, telemedicine could optimize all the steps of the HCV treatment cascade in PWUDs. If properly integrated with care services, it is able to maximize the results and can represent a winning weapon in the global fight against viral hepatitis. However, understanding the needs of this special population is the key to the success of any treatment model.

## Figures and Tables

**Table 1:** Main treatment models of HCV infection in the PWUDs population.

First Authors	References	Year of Publication	Country	Study Design	Description of the Intervention	Telemedicine-Based Models?	HCV-RNA Positive Evaluated Patients, n	HCV-RNA Positive Treated Patients, n	Linkage to Care, %	Patients Who Have Completed Treatment, n/tot (%)	Adherence to Treatment, %	Overall SVR, n/tot (%)	Reinfection Rate, n/tot (%)
**Our model**	[8]	2022 ^‡^	Italy	Observational prospective monocentric	Decentralization “patient-tailored” model at SerDs	Yes	135	135	100	129/135 (95.6)	93.4	133/135 (98.5)	1/133 (0.75)
**Talal**	[34]	2019	USA	Prospective	Decentralization model in OST program	Yes	61	45	73.8	44/45 (97.8)	10–20% missed ≥ 1 dose	42/45 (93.3)	2/42 (4.8)
**Dhiman**	[35]	2021	India	RCT	Integrated care	Yes	n.s.	2826 *	n.s.	2280/2826 (80.7)	n.e.	1398/1552 evaluated (91.1)	n.e.
**Sivakumar**	[36]	2022	USA	NRS	Minimization of face-to-face visits	Yes	35	31	88.6	31 (100)	n.e.	29/31 (93.5)	n.e.
**Grebely**	[24]	2018	Several country	Multicentre open-label phase IV trial	Electronic blister packs	No	n.s.	103	n.s.	100/103 (97.1)	94	97/103 (94.2)	1/98 (1)
**Norton**	[37]	2019	USA	NRS	Financial incentives	No	12	9	75	9/9 (100)	74	9/9 (100)	n.e.
**Ward**	[38]	2019	USA	RCT	Financial incentives	No	54	41	76	39/41 (95.1)	97.6	37/41 (90.2)	1/38 (2.6)
					Peer mentors	No	54	45	83	42/45 (93.3)	97.8	41/45 (91.1)	0/41 (0)
**Akiyama**	[39]	2019	USA	RCT	Directly observed therapy	No	n.s.	51	n.s.	50/51 (98)	86	50/51 (98)	n.e.
					Self-administered treatment		n.s.	51	n.s.	48/51 (94)	75	46/51 (90.2)	n.e.
**Messina**	[31]	2020	Italy	Prospective	Training and partial decentralization	no	n.s.	45	84	45/45 (100)	n.e.	45/45 (100)	n.e.
**Schmidbauer**	[40]	2020	Austria	n.s.	Directly observed therapy	No	n.s.	74	n.s.	74/74 (100)	94.6	70/74 (94.6)	1/70 (1.4)
**Wade**	[41]	2020	Australia/New Zealand	RCT	Primary care	No	48	43	89.6	39/43 (90.7)	n.e.	28/43 (65.1)	n.e.
					hospital-based specialist care		29	18	62.1	17/18 (94.4)	n.e.	16/18 (88.9)	n.e.
**Rinaldi**	[42]	2021	Italy	Retrospective/prospective, multicenter	SerD	No	n.s.	1460	n.s.	1451/1460 (99.4)	n.e.	1404/1460 (96.2)	n.e.
					non-SerD	No	n.s.	249	n.s.	241/249 (96.8)	n.e.	228/249 (91.6)	n.e.
**Mangia**	[43]	2021	Italy	n.s.	Training, fast-track screening, dedicated transportation service	No	231	226	97.8	220/226 (97.3)	97.7	217/220 (98.6)	1/217 (0.5)
**Byrne**	[44]	2022	Scotland	Retrospective	Community pharmacies	No	n.s.	144	n.s.	140/144 (97.2)	n.e.	131/144 (91)	12/131 (9.2)

n.e.: not evaluated; n.s.: not specified; NRS: non-randomized study; OST: opioid substitution therapy; RCT: randomized clinical trial; SerD: Italian services for addiction. * out of a total of 3477 patients, 651 treatments were still in progress at the time of drafting the text (data cannot be evaluated) ^‡^ under evaluation.

## Data Availability

Not applicable.

## References

[1] Polaris Observatory HCV Collaborators (2022). Global change in hepatitis C virus prevalence and cascade of care between 2015 and 2020: A modelling study. Lancet Gastroenterol. Hepatol..

[2] World Health Organization (2017). Global Hepatitis Report 2017.

[3] Kondili L.A., Craxì A., Aghemo A. (2021). Absolute targets for HCV elimination and national health policy paradigms: Foreseeing future requirements. Liver Int..

[4] Blach S., Kondili L.A., Aghemo A., Cai Z., Dugan E., Estes C., Gamkrelidze I., Ma S., Pawlotsky J.-M., Razavi-Shearer D. (2021). Impact of COVID-19 on global HCV elimination efforts. J. Hepatol..

[5] Nevola R., Messina V., Marrone A., Coppola N., Rescigno C., Esposito V., Sangiovanni V., Claar E., Pisaturo M., Fusco F.M. (2022). Epidemiology of HCV and HBV in a High Endemic Area of Southern Italy: Opportunities from the COVID-19 Pandemic—Standardized National Screening or One Tailored to Local Epidemiology?. Biology.

[6] Degenhardt L., Peacock A., Colledge S., Leung J., Grebely J., Vickerman P., Stone J., Cunningham E.B., Trickey A., Dumchev K. (2017). Global prevalence of injecting drug use and sociodemographic characteristics and prevalence of HIV, HBV, and HCV in people who inject drugs: A multistage systematic review. Lancet Glob. Health.

[7] Cousien A., Tran V.C., Deuffic-Burban S., Jauffret-Roustide M., Dhersin J.-S., Yazdanpanah Y. (2016). Hepatitis C treatment as prevention of viral transmission and liver-related morbidity in persons who inject drugs. Hepatology.

[8] Rosato V., Nevola R., Conturso V., Perillo P., Mastrocinque D., Pappalardo A., Le Pera T., Del Vecchio F., Claar E. (2022). Telemedicine improve HCV elimination among Italian people who use drugs: An innovative therapeutic model to increase the adherence to treatment into addiction care centers evaluated before and during the COVID-19. Biology.

[9] Rosato V., Nevola R., Conturso V., Perillo P., Le Pera T., Del Vecchio F., Claar E. (2021). Telemedicine improve HCV elimination among Italian people who use drugs: An innovative therapeutic model to increase the adherence to treatment into addiction care centers. J. Hepatol..

[10] Rashti R., Sharafi H., Alavian S.M., Moradi Y., Bolbanabad A.M., Moradi G. (2020). Systematic Review and Meta-Analysis of Global Prevalence of HBsAg and HIV and HCV Antibodies among People Who Inject Drugs and Female Sex Workers. Pathogens.

[11] Doerrbecker J., Behrendt P., Mateu-Gelabert P., Ciesek S., Riebesehl N., Wilhelm C., Steinmann J., Pietschmann T., Steinmann E. (2013). Transmission of Hepatitis C Virus among People Who Inject Drugs: Viral Stability and Association with Drug Preparation Equipment. J. Infect. Dis..

[12] Eckhardt B., Winkelstein E.R., Shu M.A., Carden M.R., McKnight C.A., Jarlais D.C.D., Glesby M.J., Marks K., Edlin B.R. (2017). Risk factors for hepatitis C seropositivity among young people who inject drugs in New York City: Implications for prevention. PLoS ONE.

[13] Larney S., Kopinski H., Beckwith C.G., Zaller N.D., Jarlais D.D., Hagan H., Rich J.D., van den Bergh B.J., Degenhardt L. (2013). Incidence and prevalence of hepatitis C in prisons and other closed settings: Results of a systematic review and meta-analysis. Hepatology.

[14] Blouin K., Leclerc P., Morissette C., Roy É., Blanchette C., Parent R., Serhir B., Alary M. (2016). Sex Work as an Emerging Risk Factor for Human Immunodeficiency Virus Seroconversion Among People who Inject Drugs in the SurvUDI Network. Sex Transm. Dis..

[15] Robaeys G., Bielen R., Azar D.G., Razavi H., Nevens F. (2016). Global genotype distribution of hepatitis C viral infection among people who inject drugs. J. Hepatol..

[16] Foster G.R., Afdhal N., Roberts S.K., Bräu N., Gane E.J., Pianko S., Lawitz E., Thompson A., Shiffman M.L., Cooper C. (2015). Sofosbuvir and Velpatasvir for HCV Genotype 2 and 3 Infection. N. Engl. J. Med..

[17] Pawlotsky J.M., Negro F., Aghemo A., Berenguer M., Dalgard O., Dusheiko G., Marra F., Puoti M., Wedemeyer H., European Association for the Study of the Liver (2020). EASL recommendations on treatment of hepatitis C: Final update of the series. J. Hepatol..

[18] Fattovich G., Stroffolini T., Zagni I., Donato F. (2004). Hepatocellular carcinoma in cirrhosis: Incidence and risk factors. Gastroenterology.

[19] Macdonald D.-C., Nelson M., Bower M., Powles T. (2008). Hepatocellular carcinoma, human immunodeficiency virus and viral hepatitis in the HAART era. World J. Gastroenterol..

[20] Marino A., Zafarana G., Ceccarelli M., Cosentino F., Moscatt V., Bruno G., Bruno R., Benanti F., Cacopardo B., Celesia B.M. (2021). Immunological and Clinical Impact of DAA-Mediated HCV Eradication in a Cohort of HIV/HCV Coinfected Patients: Monocentric Italian Experience. Diagnostics.

[21] Shih Y.-F., Liu C.-J. (2020). Hepatitis C Virus and Hepatitis B Virus Co-Infection. Viruses.

[22] Latham N.H., Doyle J.S., Palmer A.Y., Vanhommerig J.W., Agius P., Goutzamanis S., Li Z., Pedrana A., Gottfredsson M., Bouscaillou J. (2019). Staying hepatitis C negative: A systematic review and meta-analysis of cure and reinfection in people who inject drugs. Liver Int..

[23] Schwarz T., Horváth I., Fenz L., Schmutterer I., Rosian-Schikuta I., Mårdh O. (2022). Interventions to increase linkage to care and adherence to treatment for hepatitis C among people who inject drugs: A systematic review and practical considerations from an expert panel consultation. Int. J. Drug Policy.

[24] Grebely J., Dalgard O., Conway B., Cunningham E.B., Bruggmann P., Hajarizadeh B., Amin J., Bruneau J., Hellard M., Litwin A.H. (2018). Sofosbuvir and velpatasvir for hepatitis C virus infection in people with recent injection drug use (SIMPLIFY): An open-label, single-arm, phase 4, multicentre trial. Lancet Gastroenterol. Hepatol..

[25] Iversen J., Grebely J., Topp L., Wand H., Dore G., Maher L. (2014). Uptake of hepatitis C treatment among people who inject drugs attending Needle and Syringe Programs in Australia, 1999–2011. J. Viral Hepat..

[26] Messina V., Onorato L., Di Caprio G., Claar E., Iovinella V., Russo A., Rosato V., Salzillo A., Nevola R., Simeone F. (2020). Directly Acting Antiviral-Based Treatment for HCV-Infected Persons Who Inject Drugs: A Multicenter Real-Life Study. Life.

[27] Christensen S., Buggisch P., Mauss S., Böker K.H.W., Schott E., Klinker H., Zimmermann T., Weber B., Reimer J., Serfert Y. (2018). Direct-acting antiviral treatment of chronic HCV-infected patients on opioid substitution therapy: Still a concern in clinical practice?. Addiction.

[28] Ghany M.G., Morgan T.R., AASLD-IDSA Hepatitis C Guidance Panel (2020). Hepatitis C Guidance 2019 Update: American Association for the Study of Liver Diseases–Infectious Diseases Society of America Recommendations for Testing, Managing, and Treating Hepatitis C Virus Infection. Hepatology.

[29] Jordan A.E., Perlman D.C., Reed J., Smith D.J., Hagan H. (2017). Patterns and Gaps Identified in a Systematic Review of the Hepatitis C Virus Care Continuum in Studies among People Who Use Drugs. Front. Public Health.

[30] Corcorran M.A., Tsui J.I., Scott J.D., Dombrowski J.C., Glick S.N. (2021). Age and gender-specific hepatitis C continuum of care and predictors of direct acting antiviral treatment among persons who inject drugs in Seattle, Washington. Drug Alcohol Depend..

[31] Messina V., Russo A., Parente E., Russo G., Raimondo T., Salzillo A., Simeone F., Onorato L., Di Caprio G., Pisaturo M. (2020). Innovative procedures for micro-elimination of HCV infection in persons who use drugs. J. Viral Hepat..

[32] Gonzalez S.A., Fierer D.S., Talal A.H. (2017). Medical and Behavioral Approaches to Engage People Who Inject Drugs into Care for Hepatitis C Virus Infection. Addict. Disord. Their Treat..

[33] Oru E., Trickey A., Shirali R., Kanters S., Easterbrook P. (2021). Decentralisation, integration, and task-shifting in hepatitis C virus infection testing and treatment: A global systematic review and meta-analysis. Lancet Glob. Health.

[34] Talal A.H., Andrews P., McLeod A., Chen Y., Sylvester C., Markatou M., Brown L.S. (2019). Integrated, Co-located, Telemedicine-based Treatment Approaches for Hepatitis C Virus Management in Opioid Use Disorder Patients on Methadone. Clin. Infect. Dis..

[35] Dhiman R.K., Grover G.S., Premkumar M., Roy A., Taneja S., Duseja A., Arora S. (2021). Outcomes of real-world integrated HCV microelimination for people who inject drugs: An expansion of the punjab model. eClinicalMedicine.

[36] Sivakumar A., Madden L., DiDomizio E., Eller A., Villanueva M., Altice F.L. (2022). Treatment of Hepatitis C virus among people who inject drugs at a syringe service program during the COVID-19 response: The potential role of telehealth, medications for opioid use disorder and minimal demands on patients. Int. J. Drug Policy.

[37] Norton B.L., Bachhuber M.A., Singh R., Agyemang L., Arnsten J.H., Cunningham C.O., Litwin A.H. (2019). Evaluation of contingency management as a strategy to improve HCV linkage to care and treatment in persons attending needle and syringe programs: A pilot study. Int. J. Drug Policy.

[38] Ward K.M., Falade-Nwulia O., Moon J., Sutcliffe C.G., Brinkley S., Haselhuhn T., Katz S., Herne K., Arteaga L., Mehta S.H. (2019). A Randomized Controlled Trial of Cash Incentives or Peer Support to Increase HCV Treatment for Persons with HIV Who Use Drugs: The CHAMPS Study. Open Forum Infect. Dis..

[39] Akiyama M.J., Norton B.L., Arnsten J.H., Agyemang L., Heo M., Litwin A.H. (2019). Intensive Models of Hepatitis C Care for People Who Inject Drugs Receiving Opioid Agonist Therapy. Ann. Intern. Med..

[40] Schmidbauer C., Schubert R., Schütz A., Schwanke C., Luhn J., Gutic E., Pirker R., Lang T., Reiberger T., Haltmayer H. (2020). Directly observed therapy for HCV with glecaprevir/pibrentasvir alongside opioid substitution in people who inject drugs—First real world data from Austria. PLoS ONE.

[41] Wade A.J., Doyle J.S., Gane E., Stedman C., Draper B., Iser D., Roberts S.K., Kemp W., Petrie D., Scott N. (2020). Outcomes of Treatment for Hepatitis C in Primary Care, Compared to Hospital-based Care: A Randomized, Controlled Trial in People Who Inject Drugs. Clin. Infect. Dis..

[42] Rinaldi L., Messina V., Di Marco V., Iovinella V., Claar E., Cariti G., Sacco R., De Luca M., Scifo G., Gatti P. (2021). Factors Enhancing Treatment of Hepatitis C Virus–Infected Italian People Who Use Drugs: The CLEO-GRECAS Experience. Am. J. Gastroenterol..

[43] Mangia A., Rina M.F., Canosa A., Piazzolla V., Squillante M.M., Agostinacchio E., Cocomazzi G., Visaggi E., Augello N., Iannuzziello C. (2021). Increased Hepatitis C virus screening, diagnosis and linkage to care rates among people who use drugs through a patient-centered program from Italy. United Eur. Gastroenterol. J..

[44] Byrne C.J., Beer L., Inglis S.K., Robinson E., Radley A., Goldberg D.J., Hickman M., Hutchinson S., Dillon J.F. (2022). Real-world outcomes of rapid regional hepatitis C virus treatment scale-up among people who inject drugs in Tayside, Scotland. Aliment. Pharmacol. Ther..

[45] Talal A.H., McLeod A., Andrews P., Nieves-McGrath H., Chen Y., Reynolds A., Sylvester C., Dickerson S.S., Markatou M., Brown L.S. (2019). Patient Reaction to Telemedicine for Clinical Management of Hepatitis C Virus Integrated into an Opioid Treatment Program. Telemed. J. e-Health.

[46] Radley A., de Bruin M., Inglis S.K., Donnan P.T., Hapca A., Barclay S.T., Fraser A., Dillon J. (2020). Clinical effectiveness of pharmacist-led versus conventionally delivered antiviral treatment for hepatitis C virus in patients receiving opioid substitution therapy: A pragmatic, cluster-randomised trial. Lancet Gastroenterol. Hepatol..

[47] Syed T.A., Cherian R., Lewis S., Sterling R.K. (2020). Telemedicine HCV treatment in department of corrections results in high SVR in era of direct-acting antivirals. J. Viral Hepat..

[48] Cuadrado A., Cobo C., Mateo M., Blasco A.J., Cabezas J., Llerena S., I Fortea J., Lázaro P., Crespo J. (2020). Telemedicine efficiently improves access to hepatitis C management to achieve HCV elimination in the penitentiary setting. Int. J. Drug Policy.

[49] Galán G.J., Alia C.A., González M.V., Berriguete R.M.G., González F.F., Rodríguez C.M.F., Fernández M.G., García M.L.G., Losa J.E., Velasco M. (2019). The contribution of telemedicine to hepatitis C elimination in a correctional facility. Rev. Esp. Enferm. Dig..

[50] Sterling R.K., Cherian R., Lewis S., Genther K., Driscoll C., Martin K., Goode M.B., Matherly S., Siddiqui M.S., Luketic V.A. (2018). Treatment of HCV in the Department of Corrections in the Era of Oral Medications. J. Correct. Health Care.

[51] Morey S., Hamoodi A., Jones D., Young T., Thompson C., Dhuny J., Buchanan E., Miller C., Hewett M., Valappil M. (2019). Increased diagnosis and treatment of hepatitis C in prison by universal offer of testing and use of telemedicine. J. Viral Hepat..

[52] Halder A., Li V.G., Sebastian M., Nazareth S., Tuma R., Cheng W., Doyle A. (2021). Use of telehealth to increase treatment access for prisoners with chronic hepatitis C. Intern. Med. J..

[53] Papaluca T., McDonald L., Craigie A., Gibson A., Desmond P., Wong D., Winter R., Scott N., Howell J., Doyle J.S. (2019). Outcomes of treatment for hepatitis C in prisoners using a nurse-led, statewide model of care. J. Hepatol..

[54] Allah M.A., Wahed S., Ammar I., Kamal E., Alboraie M., Abdel-Razek W., Hassany M., El-Serafy M., Waked I., Doss W. (2021). Utility of telemedicine in the treatment of patients with chronic HCV infection using DAAs in remote areas with limited resources. Liver Int..

[55] Hernandez J.L.P., Mendoza R.L., Martinez J.L., Roldan J.F.T., Rosales P.A.C., Arredondo H.A.M., Gonzalez V.R., Silva L.D.L.C., Santana-Vargas D., Tijera M.D.F.H.D.L. (2021). Chronic viral hepatitis C micro-elimination program using telemedicine. The Mexican experience. Rev. Esp. Enferm. Dig..

[56] Du P., Wang X., Kong L., Jung J. (2021). Can Telementoring Reduce Urban-Rural Disparities in Utilization of Direct-Acting Antiviral Agents?. Telemed. J. e-Health.

[57] Mashru J., Kirlew M., Saginur R., Schreiber Y.S. (2017). Management of infectious diseases in remote northwestern Ontario with telemedicine videoconference consultations. J. Telemed. Telecare.

[58] Stephens D., Leston J., Terrault N.A., Gailloux K., Mera J., Essex W., Reilley B. (2019). An Evaluation of Hepatitis C Virus Telehealth Services Serving Tribal Communities: Patterns of Usage, Evolving Needs, and Barriers. J. Public Health Manag. Pract..

[59] Doica I.P., Florescu D.N., Oancea C.N., Turcu-Stiolica A., Subtirelu M.-S., Dumitra G., Rogoveanu I., Gheonea D.I., Ungureanu B.S. (2021). Telemedicine Chronic Viral Hepatitis C Treatment during the Lockdown Period in Romania: A Pilot Study. Int. J. Environ. Res. Public Health.

[60] Morales-Arraez D., Hernández-Bustabad A., Medina-Alonso M.J., Santiago-Gutiérrez L.G., García-Gil S., Diaz-Flores F., Pérez-Pérez V., Nazco J., de Rota Martin P.F., Gutiérrez F. (2021). Telemedicine and decentralized hepatitis C treatment as a strategy to enhance retention in care among people attending drug treatment centres. Int. J. Drug Policy.

[61] Cooper C.L., Hatashita H., Corsi D.J., Parmar P., Corrin R., Garber G. (2017). Direct-Acting Antiviral Therapy Outcomes in Canadian Chronic Hepatitis C Telemedicine Patients. Ann. Hepatol..

[62] Haridy J., Iyngkaran G., Nicoll A., Hebbard G., Tse E., Fazio T. (2021). eHealth Technologies for Screening, Diagnosis, and Management of Viral Hepatitis: A Systematic Review. Clin. Gastroenterol. Hepatol..

[63] Ho S.B., Bräu N., Cheung R., Liu L., Sanchez C., Sklar M., Phelps T.E., Marcus S.G., Wasil M.M., Tisi A. (2015). Integrated Care Increases Treatment and Improves Outcomes of Patients with Chronic Hepatitis C Virus Infection and Psychiatric Illness or Substance Abuse. Clin. Gastroenterol. Hepatol..

[64] Bajis S., Dore G.J., Hajarizadeh B., Cunningham E.B., Maher L., Grebely J. (2017). Interventions to enhance testing, linkage to care and treatment uptake for hepatitis C virus infection among people who inject drugs: A systematic review. Int. J. Drug Policy.

[65] Zhou K., Fitzpatrick T., Walsh N., Kim J.Y., Chou R., Lackey M., Scott J., Lo Y.-R., Tucker J.D. (2016). Interventions to optimise the care continuum for chronic viral hepatitis: A systematic review and meta-analyses. Lancet Infect. Dis..

[66] Socías M.E., Karamouzian M., Parent S., Barletta J., Bird K., Ti L. (2019). Integrated models of care for people who inject drugs and live with hepatitis C virus: A systematic review. Int. J. Drug Policy.

[67] Delile J.-M., De Ledinghen V., Jauffret-Roustide M., Roux P., Reiller B., Foucher J., Dhumeaux D. (2018). Hepatitis C virus prevention and care for drug injectors: The French approach. Hepatol. Med. Policy.

[68] Coffin P.O., Santos G.-M., Behar E., Hern J., Walker J., Matheson T., Kinnard E.N., Silvis J., Vittinghoff E., Fox R. (2019). Randomized feasibility trial of directly observed versus unobserved hepatitis C treatment with ledipasvir-sofosbuvir among people who inject drugs. PLoS ONE.

[69] McDermott C.L., Lockhart C.M., Devine B. (2018). Outpatient directly observed therapy for hepatitis C among people who use drugs: A systematic review and meta-analysis. J. Virus Erad..

[70] Surey J., Menezes D., Francis M., Gibbons J., Sultan B., Miah A., Abubakar I., Story A. (2019). From peer-based to peer-led: Redefining the role of peers across the hepatitis C care pathway: HepCare Europe. J. Antimicrob. Chemother..

